# Vital Signs: Restraint Use and Motor Vehicle Occupant Death Rates Among Children Aged 0–12 Years — United States, 2002–2011

**Published:** 2014-02-07

**Authors:** Erin K. Sauber-Schatz, Bethany A. West, Gwen Bergen

**Affiliations:** 1 Division of Unintentional Injury Prevention, National Center for Injury Prevention and Control, CDC

## Abstract

**Background:**

Motor vehicle crashes are a leading cause of death among children in the United States. Age- and size-appropriate child restraint use is the most effective method for reducing these deaths.

**Methods:**

CDC analyzed 2002–2011 data from the Fatality Analysis Reporting System to determine the number and rate of motor-vehicle occupant deaths, and the proportion of unrestrained child deaths among children aged <1 year, 1–3 years, 4–7 years, 8–12 years, and for all children aged 0–12 years. Age group–specific death rates and proportions of unrestrained child motor vehicle deaths for 2009–2010 were further stratified by race/ethnicity.

**Results:**

Motor vehicle occupant death rates for children declined significantly from 2002 to 2011. However, one third (33%) of children who died in 2011 were unrestrained. Compared with white children for 2009–2010, black children had significantly higher death rates, and black and Hispanic children both had significantly higher proportions of unrestrained child deaths.

**Conclusions:**

Motor vehicle occupant deaths among children in the United States have declined in the past decade, but more deaths could be prevented if restraints were always used.

**Implications for Public Health:**

Effective interventions, including child passenger restraint laws (with child safety seat/booster seat coverage through at least age 8 years) and child safety seat distribution plus education programs, can increase restraint use and reduce child motor vehicle deaths.

## Introduction

Motor vehicle crashes are a leading cause of death among children in the United States (1). Child safety seat (CSS) use reduces the risk for death to infants (aged <1 year) by 71%; and to toddlers (aged 1–4 years) by 54% in passenger vehicles (2,3). Booster seat use reduces the risk for serious injury by 45% for children aged 4–8 years when compared with seat belt use alone (4). For older children and adults, seat belt use reduces the risk for death and serious injury by approximately half (5). Based on this evidence, CDC recommends using age- and size-appropriate child restraints (including CSS and booster seats) in the back seat until adult seat belts fit properly (i.e., when the lap belt lays across the upper thighs, not the stomach; and the shoulder belt lays across the shoulder and chest, not the neck or face), which normally occurs after a child is at least age 8 years or ≥57 inches (145 cm) tall (6). The Community Preventive Services Task Force recommends CSS laws and CSS distribution plus education programs based on strong evidence of their effectiveness for increasing restraint use and decreasing injuries and deaths to child passengers (7). Distribution plus education programs are also recommended in a more recent review for increasing restraint use (8). The purpose of this study was to explore data over the past decade on child motor vehicle occupant deaths, determine the proportion of unrestrained child deaths, and explore differences by age, sex, and race/ethnicity.

## Methods

For this study, CDC used Fatality Analysis Reporting System data, which include motor vehicle crashes that occur on public roads in the United States in which at least one vehicle occupant or nonoccupant (pedestrian, bicyclist, etc.) involved in the crash dies within 30 days. Deaths among motor vehicle occupants aged 0–12 years in passenger vehicles (i.e., passenger cars, pickup trucks, vans, and sport utility vehicles) during 2002–2011 were included in this study. Analyses were conducted among all children aged 0–12 years and were stratified into the following four age groups: <1 year, 1–3 years, 4–7 years, and 8–12 years; coinciding with the recommended ages for the various types of child restraints during the study period. Population counts were obtained from the U.S. Census Bureau for the same age groups and years.

Annual motor vehicle occupant death rates per 100,000 population were calculated for 2002–2011. The percent changes in death rates were calculated over the past decade. Age group–specific death rates for 2009–2010 (the most recent years of finalized race/ethnicity data available at the time of study analyses) were further stratified by sex and race/ethnicity. The proportion of motor vehicle deaths that involved children who were unrestrained (hereafter referred to as the proportion of unrestrained child deaths) were calculated for 2002–2011 by dividing the number of unrestrained deaths by all child motor vehicle occupant deaths, including deaths for which restraint use status was unknown.

Proportions of unrestrained child deaths were calculated by age group and race/ethnicity. The percentage changes in the proportions of unrestrained child deaths were calculated over the past decade. To account for small numbers, 2 years of data were combined at the beginning and end of the decade (2002–2003 and 2009–2010) for race/ethnicity percent change calculations. Race/ethnicity was divided into five mutually exclusive categories: non-Hispanic whites, blacks, American Indians/Alaska Natives (AI/AN), Asian/Pacific Islanders (A/PI), and Hispanics of all races. However, because AI/ANs and A/PIs had <20 deaths in each age group, they were not included in the racial stratification analyses.

Death rate standard errors were calculated by dividing the death rate by the square root of the number of deaths (9). Normal approximation was used to calculate 95% confidence intervals for rates when the number of deaths was ≥100 and Poisson approximation was used when the number of deaths was <100 (9). Poisson distribution was used to calculate standard errors for proportions of unrestrained child deaths and U.S. Census Bureau methods were used to calculate 95% confidence intervals for percentage changes (10).

## Results

**FIGURE 1:**
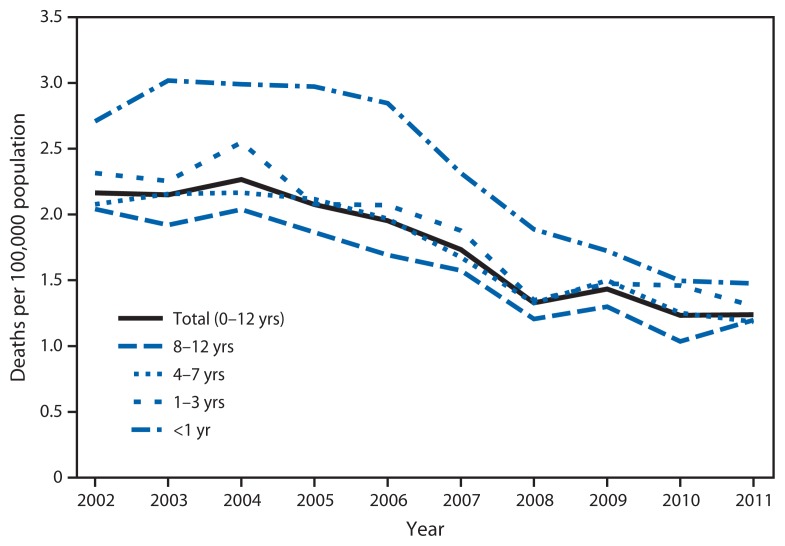
Motor vehicle occupant deaths per 100,000 population for children aged 0–12 years, by age group and year — United States, 2002–2011

**FIGURE 2:**
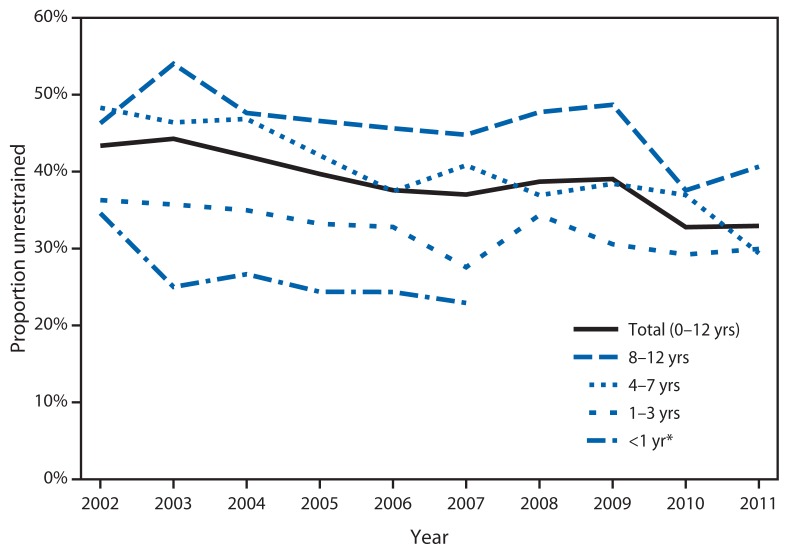
Proportion of unrestrained child motor vehicle deaths by age group and year — United States, 2002–2011 * 2008–2011 unrestrained deaths for children aged <1 years not shown because annual counts were <20.

During 2002–2011, a total of 9,182 children aged 0–12 years died in motor vehicle crashes in the United States. During this period, motor vehicle death rates among children aged 0–12 years decreased 43%, from 2.2 deaths per 100,000 population in 2002 to 1.2 in 2011 (Figure 1). By age group, motor vehicle death rates decreased significantly among children aged <1 year by 45% (2.7 to 1.5 per 100,000 population), 1–3 years by 44% (2.3 to 1.3 per 100,000 population), 4–7 years by 43% (2.1 to 1.2 per 100,000 population), and 8–12 years by 41% (2.0 to 1.2 per 100,000 population). Also during 2002–2011, the proportion of unrestrained child deaths decreased significantly for children aged 1–3 years (by 18%), aged 4–7 years (by 39%), and aged 0–12 years (by 24%) (Figure 2). However, in 2011, 33% of children aged 0–12 years who died as occupants of motor vehicles were unrestrained.

During 2009–2010, a total of 1,409 children aged 0–12 years died in motor vehicle crashes, a rate of 1.3 deaths per 100,000 population (Table). Death rates did not differ significantly by sex or age group, but did differ by race. Black children had significantly higher death rates than white children among those aged 1–3 years (2.0 versus 1.0 deaths per 100,000 population) and for all children aged 0–12 years combined (1.5 versus 1.0 deaths per 100,000 population). Additionally, black children had a significantly higher proportion of unrestrained child deaths compared with white children for those aged 1–3 years (47% versus 20%), 4–7 years (46% versus 26%), and for all children aged 0–12 years combined (45% versus 26%). Although no significant differences in motor vehicle death rates were found for Hispanic children compared with white children, Hispanic children had a significantly higher proportion of unrestrained child deaths compared with white children for those aged 4–7 years (50% versus 26%), 8–12 years (55% versus 33%), and 0–12 years (46% versus 26%).

From 2002–2003 to 2009–2010, the proportion of unrestrained child deaths decreased significantly among children aged 0–12 years, by 27% for whites, 16% for blacks, and 14% for Hispanics. Unrestrained child deaths also decreased, by 26% and 29% among white children aged 4–7 years and 8–12 years, respectively, by 28% among black children aged 4–7 years, and by 36% among Hispanic children aged 1–3 years.

## Conclusions and Comment

This study found that child motor vehicle occupant death rates and the proportion of unrestrained child deaths decreased from 2002 to 2011. However, this study also found that >9,000 child motor vehicle occupants died during 2002–2011, and in 2011, still one third of children who died were unrestrained. During a motor vehicle crash, age- and size-appropriate restraint use is the most effective way to prevent injuries and deaths (5).

Compared with the relatively low proportion of unrestrained children seen in observational studies (11,12), the proportion of unrestrained child deaths is much higher. Among child passengers aged <1 year in 2011, for example, 2% were observed to be unrestrained (11), but 22% of children in that age group who died in motor vehicle crashes were unrestrained (proportion based on known restraint use) (5). The known effectiveness of restraints, coupled with the overrepresentation of unrestrained child deaths, demonstrates that more child motor vehicle deaths could be prevented through increased child restraint use. Based on National Highway Traffic Safety Administration calculations, an estimated 3,308 lives were saved by CSS use among children aged 0–4 years during 2002–2011. If CSSs were used in motor vehicles 100% of the time for children aged 0–4 years, an additional 837 lives could have been saved (Marc Starnes, National Highway Traffic Safety Administration, personal communication, 2013) (13).

This study found that black children had the highest rates of motor vehicle occupant death compared with whites and Hispanics for children aged 1–3 years and aged 0–12 years combined. Racial/ethnic groups with the highest death rates also had higher proportions of unrestrained child deaths. A previous analysis of 2006 Fatality Analysis Reporting System data found that blacks had the highest proportion of unrestrained child deaths (52%), followed closely by Hispanics (51%) (proportion based on known restraint use) (14). The current study confirmed this, with blacks and Hispanics having a higher proportion of unrestrained child deaths than whites. In addition, this racial/ethnic difference in restraint use is found in observational studies and injury data, with black children more likely to be unrestrained than white children, and in self-reported data, with black and Hispanic children more likely to be unrestrained than white children (15–17). Socio-economic status might be a contributing factor to racial/ethnic differences. In a study of trauma patients, children insured with Medicaid were more likely to be black, and were less likely to be restrained than those with private insurance, suggesting that economically disadvantaged children might be less likely to be restrained (17).

Although observed restraint use increased from 88% in 2002 to 91% in 2011 among children aged 0–7 years (12), changes in observed restraint use varied by race/ethnicity. From 2006 to 2011, observed restraint use for white children aged 1–12 years increased or stayed the same (99% to 99% for ages 1–3 years, 93% to 96% for ages 4–7 years, and 85% to 91% for ages 8–12 years), while it decreased for Hispanic children of the same age (93% to 90% for ages 1–3 years, 92% to 79% for ages 4–7 years, and 84% to 83% for ages 8–12 years). During this period, observed restraint use for black children increased among those aged 1–7 years (89% to 90% for ages 1–3 years; 74% to 84% for ages 4–7 years), but decreased for among those aged 8–12 years (79% to 76%) (11,18). Further research is needed to better explore and understand these racial/ethnic differences.

Previous research found that child restraint use also differs by age, with the highest use among the youngest children (11). In a 2011 survey, children aged <1 year had observed restraint use of 98%; whereas, children aged 8–12 years had observed restraint use of 88% (11). Similarly, previous research found that among child motor vehicle deaths aged 12 years and younger the proportion of unrestrained child deaths increased with age. Specifically, children aged <1 year had the lowest proportion (22%) of unrestrained child deaths, followed by children aged 1–4 years (32%) and aged 4–7 years (34%), based on known restraint use. Children aged 8–12 years had the highest proportion of unrestrained child deaths (45%), based on known restraint use (5,19). The current study confirmed this trend.

Effective interventions can increase restraint use among child motor vehicle occupants and prevent associated deaths and injuries. A Community Preventive Services Task Force systematic review found that CSS laws decrease deaths by a median of 35% and increase CSS use by a median of 13%, and CSS distribution plus education programs increase CSS possession by a median 51% and CSS use by a median of 23% (7). Based on these findings and strong evidence of effectiveness, the Task Force recommends both of these interventions to increase restraint use and reduce deaths. Increasing the required age for CSS/booster seat use in state child passenger restraint laws is also an effective way to increase restraint use among older children. A recent study of five states that increased the age requirement to 7 or 8 years for CSS/booster seat use found that the rate of children using CSS/booster seats increased nearly three times and the rate of children who sustained fatal or incapacitating injuries decreased by 17% (20).

**FIGURE 3:**
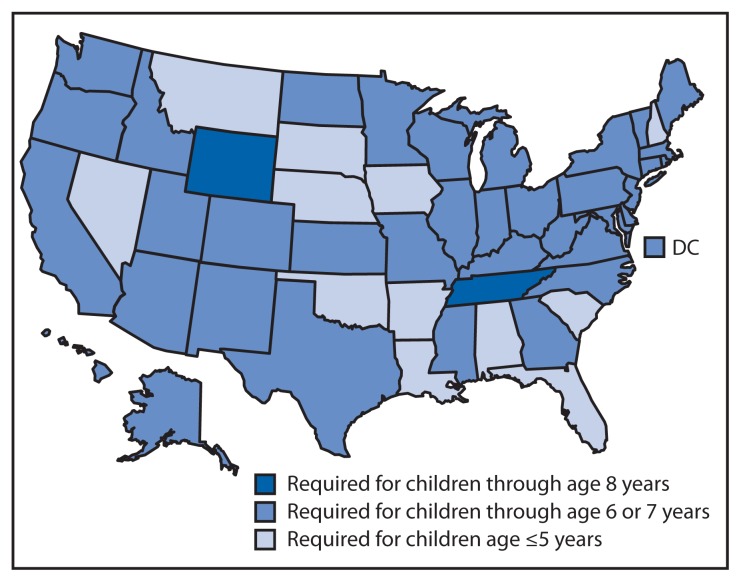
Child passenger restraint laws requiring use of child safety or booster seats, by age requirement and state* — United States, August 2013 * Only age was used to determine child passenger restraint law coverage. Some state also have specific height and/or weight requirements.

Since 2002, a majority of states have increased the required age for CSS/booster seat use. However, in 2013, 12 states had child passenger restraint laws that required CSS/booster seat use by children aged ≤5 years; 36 states and the District of Columbia had laws requiring CSS/booster seats use by children through either age 6 or 7 years; and two states (Tennessee and Wyoming) had laws requiring CSS/booster seat use by children through at least age 8 years. As a result, in 2013, only 2% of children in the United States lived in states with a child passenger restraint law that required CSS/booster seat use by children through at least age 8 years (Figure 3).

Motor vehicle traffic death rates for children are higher in the United States than in other high income countries. In 2011, motor vehicle traffic death rates among children aged ≤14 years were below the U.S. rate (1.9 deaths per 100,000 population) in the United Kingdom (0.5), Sweden (0.6), Italy (0.7), Germany (0.8), Norway (0.9), and Canada (1.1). Notably, the child motor vehicle occupant death rate per 100,000 population in the United States is more than double that of 22 selected high-income European countries combined (1.9 versus 0.9 per 100,000 population, respectively) (21).

The findings in this report are subject to at least three limitations. First, Fatality Analysis Reporting System data are extracted from police reports of motor vehicle crashes and death certificates rather than self reports; therefore, some racial/ethnic misclassification is likely. Additionally, 13% of deaths (n = 182) from 2009–2010 had unknown race/ethnicity and were excluded from racial stratification analyses. Second, the reported proportions of unrestrained child deaths are likely underestimates; the proportion of deaths that had unknown restraint use ranged from a low of 7% in 2007 to a high of 29% in 2010. Finally, other factors, such as safer cars, safer child safety/booster seats, and the economy, might have contributed to the decrease in child motor vehicle occupant death rates. This study was not able to account for changes in these factors.

To reduce the number of child motor vehicle occupant deaths, parents and caregivers should ensure that children always travel in the back seat in age- and size-appropriate restraints as follows: rear-facing CSSs up to age 2 years; forward-facing CSSs up to at least age 5 years; booster seats through at least age 8 years and until seat belts fit properly; and adult seat belts, still in the back seat, until age 13 years. Passengers aged ≥13 years should use adult seat belts on every trip. Implementing interventions that are proven to increase child restraint use is an effective way to prevent child motor vehicle injuries and deaths. These interventions include child passenger restraint laws that require CSS/booster seat use in the back seat until a child is aged ≥8 years and CSS distribution plus education programs.

**Key Points**

- Motor vehicle occupant death rates among children aged 0–12 years decreased by 43% from 2002 to 2011.
- Despite this decrease, one third of child motor-vehicle occupants (aged 0–12 years) who died in 2011 were unrestrained.
- Almost half of black (45%) and Hispanic (46%) children who died in crashes were unrestrained, compared with 26% of whites (2009–2010).
- In 2013, only two states had child passenger restraint laws that required child safety seat/booster seat use by children through at least age 8 years. Implementing effective interventions, such as increasing the state-required age for child safety seat/booster seat use in child passenger restraint laws and child safety seat distribution plus education programs, can reduce motor vehicle occupant deaths.
- Additional information is available at http://www.cdc.gov/vitalsigns.

## Figures and Tables

**TABLE:** Motor vehicle occupant deaths per 100,000 population for children aged 0–12 years, by selected characteristics — United States, 2009–2010*

Age group (yrs)	Characteristic	No. of deaths	Deaths per 100,000 population	(95% CI)
**<1**	**Total**	**128**	**1.6**	**(1.3–1.9)**
	Sex			
	Male	75	1.8	(1.5–4.2)
	Female	52	1.3	(1.0–3.1)
	Race/Ethnicity			
	White, non-Hispanic	53	1.3	(0.9–2.9)
	Black, non-Hispanic	22	1.8	(1.1–4.5)
	Hispanic	27	1.3	(0.9–3.2)
**1–3**	**Total**	**358**	**1.5**	**(1.3–1.6)**
	Sex			
	Male	177	1.4	(1.2–1.6)
	Female	181	1.5	(1.3–1.7)
	Race/Ethnicity			
	White, non-Hispanic	132	1.0	(0.8–1.2)
	Black, non-Hispanic	76	2.0	(1.6–4.6)
	Hispanic	68	1.1	(0.9–2.5)
**4–7**	**Total**	**445**	**1.4**	**(1.2–1.5)**
	Sex			
	Male	228	1.4	(1.2–1.6)
	Female	217	1.4	(1.2–1.6)
	Race/Ethnicity			
	White, non-Hispanic	203	1.1	(1.0–1.3)
	Black, non-Hispanic	63	1.3	(1.0–3.0)
	Hispanic	84	1.1	(0.9–2.4)
**8–12**	**Total**	**478**	**1.2**	**(1.1–1.3)**
	Sex			
	Male	248	1.2	(1.0–1.3)
	Female	230	1.1	(1.0–1.3)
	Race/Ethnicity			
	White, non-Hispanic	207	0.9	(0.8–1.0)
	Black, non-Hispanic	77	1.2	(1.0–2.8)
	Hispanic	103	1.1	(0.9–1.4)
**Total (0–12)**	**Total**	**1,409**	**1.3**	**(1.3–1.4)**
	Sex			
	Male	728	1.3	(1.3–1.4)
	Female	680	1.3	(1.2–1.4)
	Race/Ethnicity			
	White, non-Hispanic	595	1.0	(0.9–1.1)
	Black, non-Hispanic	238	1.5	(1.3–1.7)
	Hispanic	282	1.1	(1.0–1.3)

**Abbreviation:** CI = confidence interval.

* The most recent 2 years for which race/ethnicity data have been finalized at the time of analysis.
